# *Paramyxovirus* Infection Regulates T Cell Responses by BDCA-1^+^ and BDCA-3^+^ Myeloid Dendritic Cells

**DOI:** 10.1371/journal.pone.0099227

**Published:** 2014-06-11

**Authors:** Meera R. Gupta, Deepthi Kolli, Claudio Molteni, Antonella Casola, Roberto P. Garofalo

**Affiliations:** 1 Department of Internal Medicine, University of Texas Medical Branch, Galveston, Texas, United States of America; 2 Department of Pediatrics, University of Texas Medical Branch, Galveston, Texas, United States of America; 3 Department of Microbiology and Immunology, University of Texas Medical Branch, Galveston, Texas, United States of America; 4 Sealy Center for Molecular Medicine, University of Texas Medical Branch, Galveston, Texas, United States of America; 5 Pediatric Highly Intensive Care Unit, Department of Pathophysiology and Transplantation, Università degli Studi di Milano, Fondazione IRCCS Ca’ Granda Ospedale Maggiore Policlinico, Milan, Italy; University of North Carolina at Chapel Hill, United States of America

## Abstract

Respiratory syncytial virus (RSV) and human Metapneumovirus (hMPV), viruses belonging to the family *Paramyxoviridae*, are the most important causes of lower respiratory tract infection in young children. Infections with RSV and hMPV are clinically indistinguishable, and both RSV and hMPV infection have been associated with aberrant adaptive immune responses. Myeloid Dendritic cells (mDCs) play a pivotal role in shaping adaptive immune responses during infection; however, few studies have examined how interactions of RSV and hMPV with individual mDC subsets (BDCA-1^+^ and BDCA-3^+^ mDCs) affect the outcome of anti-viral responses. To determine whether RSV and hMPV induce virus-specific responses from each subset, we examined co-stimulatory molecules and cytokines expressed by BDCA-1^+^ and BDCA-3^+^ mDCs isolated from peripheral blood after infection with hMPV and RSV, and examined their ability to stimulate T cell proliferation and differentiation. Our data show that RSV and hMPV induce virus-specific and subset-specific patterns of co-stimulatory molecule and cytokine expression. RSV, but not hMPV, impaired the capacity of infected mDCs to stimulate T cell proliferation. Whereas hMPV-infected BDCA-1^+^ and BDCA-3^+^ mDCs induced expansion of Th17 cells, in response to RSV, BDCA-1^+^ mDCs induced expansion of Th1 cells and BDCA-3^+^ mDCs induced expansion of Th2 cells and Tregs. These results demonstrate a virus-specific and subset-specific effect of RSV and hMPV infection on mDC function, suggesting that these viruses may induce different adaptive immune responses.

## Introduction

Respiratory Syncytial Virus (RSV) represents the most important cause of acute lower respiratory tract infection (LRTI) in infants, young children, elderly, and immunocompromised individuals worldwide and is associated with significant short- and long-term morbidity including bronchiolitis, pneumonia, and abnormal pulmonary functions. Infection with human Metapneumovirus (hMPV) is second only to RSV as a cause of LRTI in early childhood. Infections with RSV and hMPV are clinically indistinguishable with overlapping spectra of disease symptoms. Moreover, both RSV and hMPV can cause wheezing and exacerbations of asthma in children and adults [1]–[3] and severe disease has been linked to the development of asthma and atopy [4]–[9]. RSV and hMPV are single-stranded RNA viruses belonging to the *Paramyxoviridae* family, and despite similarities in their genomic organization, two major differences do exist: hMPV lacks the nonstructural proteins NS1 and NS2 and the gene order is different [10].

Resolution of infection with these viruses is likely mediated by a similar array of immune mechanisms involving innate and cellular immunity, and the lack of long-lasting protection against reinfection and propensity for severe disease in susceptible individuals suggests that both RSV and hMPV have the ability to suppress or subvert host adaptive immune responses. Severe RSV infection has been associated with skewing the Th1/Th2 balance of the virus-specific response away from antiviral Th1 responses towards Th2 [11]–[13]. Similar findings in atopy and asthma suggest that Th2 responses induced during RSV infection may have an important pathophysiological role in the development of wheezing and asthma. Additionally, studies in murine models of acute RSV infection indicate that Tregs play an important role in determining the balance between effective antiviral immunity and controlling harmful immunopathology in the host response against RSV [14], [15]. Recent data also implicates Th17 lymphocytes as being important contributors to both the protective immune responses [16], [17], as well as the pathology associated with RSV infection [18]. The cellular response during hMPV infection in patients is not well described, although findings in murine models suggest that hMPV also induces aberrant T cell responses [19]. The mechanisms by which each virus is able to modulate the host immune response have not been fully elucidated, however, dendritic cells (DCs), as key regulators of immunity, are an ideal target for the virus to exert its immune altering mechanisms.

DCs are regarded as the most potent professional antigen presenting cell (APC) type and are an important first line of defense against invading pathogens. Upon antigen recognition, DCs produce cytokines and co-stimulatory signals needed to guide T cell differentiation, ultimately determining the quality and quantity of the resulting immune response. Different populations of human DCs are defined based on their lineage and expression of unique Blood Dendritic Cell Antigens (BDCA). Plasmacytoid DCs (pDCs) mediate antiviral immunity via the production of IFN-α and are characterized by expression of BDCA-2 [20]. Myeloid DCs (mDCs), particularly efficient in the uptake, processing and presentation of antigens, are further subdivided into functionally distinct subsets identified by differential expression of either BDCA-1 (BDCA-1^+^) or BDCA-3 (BDCA-3^+^) [21]–[24]. The DC network in the airway mucosa is comprised mainly of mDCs, with BDCA-3^+^ mDCs predominating [25], [26], and during respiratory infections, both tissue-resident and recruited DCs are activated as part of the host immune response [27]. While the relationship between mDCs found in the lung and peripheral blood is not clear, parallel phenotypic analysis and transcriptome mapping provides evidence that lung and other non-lymphoid tissue mDC subsets are phenotypically and functionally related to mDC subsets found in the circulation [28]. The strategic localization of DCs at the site of pathogen entry makes them particularly susceptible to initial viral invasion, thus studying the interactions of DCs with viruses and how this may influence the resulting immune response is critical for understanding disease pathogenesis and immunity to viral infections.

Much of what we know about the effects of RSV and hMPV infection on DC function comes from studies using murine DCs or mDCs derived *in* *vitro* from human monocytes (Mo-DCs). Studies with Mo-DCs have shown a differential response to infection with RSV and hMPV, suggesting that RSV and hMPV may use distinct mechanisms to interfere with host immune responses. However, although Mo-DCs have many characteristics similar to primary myeloid blood DCs, studies have not shown direct functional correlations between *in* *vitro* derived Mo-DCs and individual mDC subsets isolated from lymph nodes or blood [22], [29], [30]. Furthermore, Mo-DCs are unable to give rise to cells that are phenotypically or functionally equivalent to BDCA-3^+^ mDCs [22]. Thus, studies using Mo-DCs may not adequately recapitulate the function of diverse human mDC subsets during infection.

We have recently described a subset-specific effect of RSV on the functional response of BDCA-1^+^ and BDCA-3^+^ mDCs [31]. Findings of differential responses as compared to stimulation with the TLR3 agonist Poly I:C also suggests a virus-specific effect of RSV infection on BDCA-1^+^ and BDCA-3^+^ mDC function, although comparisons with other viruses was not made [31]. Specifically, the effect of hMPV infection on the functional response of primary mDC subsets has not been examined. Therefore, to identify the specific responses of BDCA-1^+^ and BDCA-3^+^ mDCs to hMPV infection and to determine whether RSV and hMPV induce virus-specific responses from each subset, we examined co-stimulatory marker expression and cytokine production by donor-matched BDCA-1^+^ and BDCA-3^+^ mDCs after infection with hMPV and RSV. The functional response after infection was further evaluated by examining their ability to stimulate CD4^+^ T cell proliferation and differentiation in a mixed lymphocyte reaction.

## Materials and Methods

### Ethics Statement

This study was approved by UTMB’s Institutional Review Board.

### Isolation of mDC Subsets

Buffy coats prepared by the University of Texas Medical Branch (UTMB) blood blank from healthy adult donors who fulfilled criteria for blood donations were obtained. The donor’s identity, race, and age remained anonymous to investigators. The isolation of mDC subsets from buffy coats has been previously described [31]. Briefly, peripheral blood mononuclear cells (PBMCs) were separated from buffy coats by Ficoll-hypaque density centrifugation and enriched for mDCs using an mDC cell isolation kit (Miletnyi Biotec, Auburn, CA) to deplete magnetically labeled non-mDCs. In preparation for FACS sorting, the DC enriched fraction was stained with the following fluorochrome-conjugated monoclonal antibodies (mAbs[clone]): PE-BDCA-3[1A4], APC-BDCA-1[AD5-8E7], FITC-BDCA-2[AC144], and FITC-lineage marker cocktail containing CD3[SK7], CD14[MΦP9], CD16[3G8], CD19[SJ25C1], CD20[L27], CD56[NCAM16.2] purchased from eBiosciences (San Diego, CA), Miltenyi Biotec, and BD Biosciences (San Jose, CA) respectively. Cells were stained in the presence of FcR-blocking reagent (Miltenyi Biotec) to minimize non-specific binding. Gating strategy for the identification and sorting of BDCA-1^+^ and BDCA-3^+^ mDCs has been previously described [31]. BDCA-3^+^ mDCs were sorted as FITC^−^, APC^−^, PE^hi^ and BDCA-1^+^ mDCs were sorted as FITC^−^, APC^+^, PE^dim^. Routine post-sort analysis showed purity ≥95%. Cell sorting was performed on a FACSAria (BD Biosciences) capable of 9-color analysis running FACS Diva software.

### Virus Preparation and Infection

Preparation of sucrose-purified RSV (Long strain) and hMPV (CAN97-83) have been previously described [32]. For infection, 1×10^4^ cells of donor-matched BDCA-1^+^ or BDCA-3^+^ mDCs were resuspended in 200 µl of cRPMI (RPMI 1640 medium +2 mmol/liter L-glutamine +2% FBS+50 µM 2-ME+100 UI/mL penicillin-streptomycin) and incubated for 40 hours at 37°C with RSV or hMPV at a multiplicity of infection (MOI) of 5 [32], [33]. For hMPV-inoculated cells, the medium also contained 1 µg/ml of trypsin during the infection process. Uninfected cells served as negative controls. When indicated, mDCs were also incubated with ultraviolet (UV)-inactivated RSV and hMPV (non-replicating virus) as additional controls.

### FACS Analysis of DCs

To detect intracellular expression of RSV and hMPV antigens, BDCA-1^+^ and BDCA-3^+^ mDCs were fixed with Cytofix/Cytoperm (Pharmingen), permeabilized with Perm/Wash buffer (Pharmingen), and stained with a monoclonal FITC-conjugated anti-RSV antibody (Biosource, Camarillo, CA) or with guinea pig anti-hMPV Ab, followed by a polyclonal FITC-goat anti-guinea pig Ab (Zymed Laboratories, Carlsbad, CA) as previously described [32]. Viability of infected cells was determined by first staining with Fixable viability dye eFlour780 (eBioscience). For co-stimulatory molecule analysis, mDCs were stained with FITC-PD-L1[M1H1], v450-CD86[2331(FUN-1)], and PE-Cy7-CD80[L307.4] (BD Biosciences). Flow cytometric data acquisition for all studies was performed on a BD LSRII Fortessa capable of 18-color analysis running FACS Diva software (BD Biosciences). FloJo V7.6.3 software was used to analyze all flow cytometry data.

### Measurement of Cytokine and IFN-α Production

After 40 hours, cell free supernatants were collected and tested for cytokines IL-1β, IL-1rα, IL-6, IL-7, IL-8/CXCL8, IL-10, IL-12(p70), IP-10/CXCL10, G-CSF, MCP-1/CCL2, MIP-1α/CCL3, MIP-1β/CCL4, RANTES/CCL5, and TNF-α, using the Luminex-based Bio-Plex system (Bio-Rad Laboratories, Hercules, CA). The lower limit of detection for all cytokines measured in this assay is 3 pg/ml. When comparing cytokine production between subsets, the fold change in concentration (infected/uninfected) was used to normalize results across cell types. To measure the production of IFN-α, the cell-free supernatant was collected at 24 hours. Human IFN-α concentration was determined by commercial ELISA assays according to the manufacturer’s instructions (PBL, Piscataway, NJ).

### CD4^+^ T Cell Proliferation Assay

Allogeneic CD4^+^ T cells were isolated from PBMCs by negative immunomagnetic selection using a CD4^+^ T cell isolation kit (Miletnyi Biotec, Auburn, CA). Routine post-sort purity was >96% as determined by flow cytometry. To track proliferation, T cells were labeled with 10 µM CFSE (Invitrogen, Grand Island, NY) according to the manufacturer’s instructions. Donor-matched BDCA-1^+^ and BDCA-3^+^ mDCs were incubated at 37°C with RSV, hMPV, UV-RSV, or UV-hMPV. After 24 hours, cells were washed and co-cultured with the CFSE-labeled allogeneic T cells at a ratio of 1∶5 in RPMI containing 5% FCS for 7 days at 37°C. T cells alone and T cells incubated with soluble CD3 and CD28 were included as controls. Prior to flow cytometry, cells were stained with a fixable viability dye eFlour 780 (eBioscience), PerCP-Cy 5.5-CD3[SK7], and PE-Cy 7-CD4[RPT-A] (BD Biosciences). Proliferation of CD3^+^ CD4^+^ T cells was measured by the loss of CFSE dye that occurs with cell division.

### CD4^+^ T Cell Differentiation Assay

For T cell differentiation studies, donor-matched BDCA-1^+^ and BDCA-3^+^ mDCs were incubated for 24 hours at 37°C with RSV, UV-RSV, hMPV, or UV-hMPV. After 24 hours, cells were washed and co-cultured with allogeneic CD4^+^ T cells at a ratio of 1∶5 in RPMI containing 5% FCS for 8 days at 37°C. On day 8, cells were harvested and stained with fixable viability dye eFlour 780 (eBioscience) to discriminate between live and dead cells, PE-Cy 7-CD4[RPT-A], and BV 605-CD25[2A3] (BD Biosciences) according to the manufacturer’s instructions. The cells were fixed and permeabilized according to the manufacturer’s instructions using the Foxp3 Transcription Factor Staining Buffer Set (eBiosciences) and immunostained with Fastimmune FITC-IFN-γ[25723.11]/PE-IL-4[3010.211] (BD Biosciences), APC-Foxp3[PCH101] (eBioscience), PerCP-Cy5.5-IL-17A [eBio64DEC17] (eBioscience).

### Statistical Analysis

Statistical Analysis was performed with InStat 5.02 biostatistics package (GraphPad Software, San Diego, CA) using one-way repeated measures analysis of variance (ANOVA) with Tukey post-hoc tests or paired t-tests to ascertain differences in the responses with a given subtype, and two-way ANOVA with Bonferroni post hoc tests were used to ascertain differences between cell types. Significance was defined as p≤0.05. Prior to analysis, data sets were log transformed to normalize non-normally distributed data. Data are reported as non-normalized values.

## Results

### BDCA-1^+^ and BDCA-3^+^ mDCs are Permissive to Infection with hMPV

**Figure 1:**
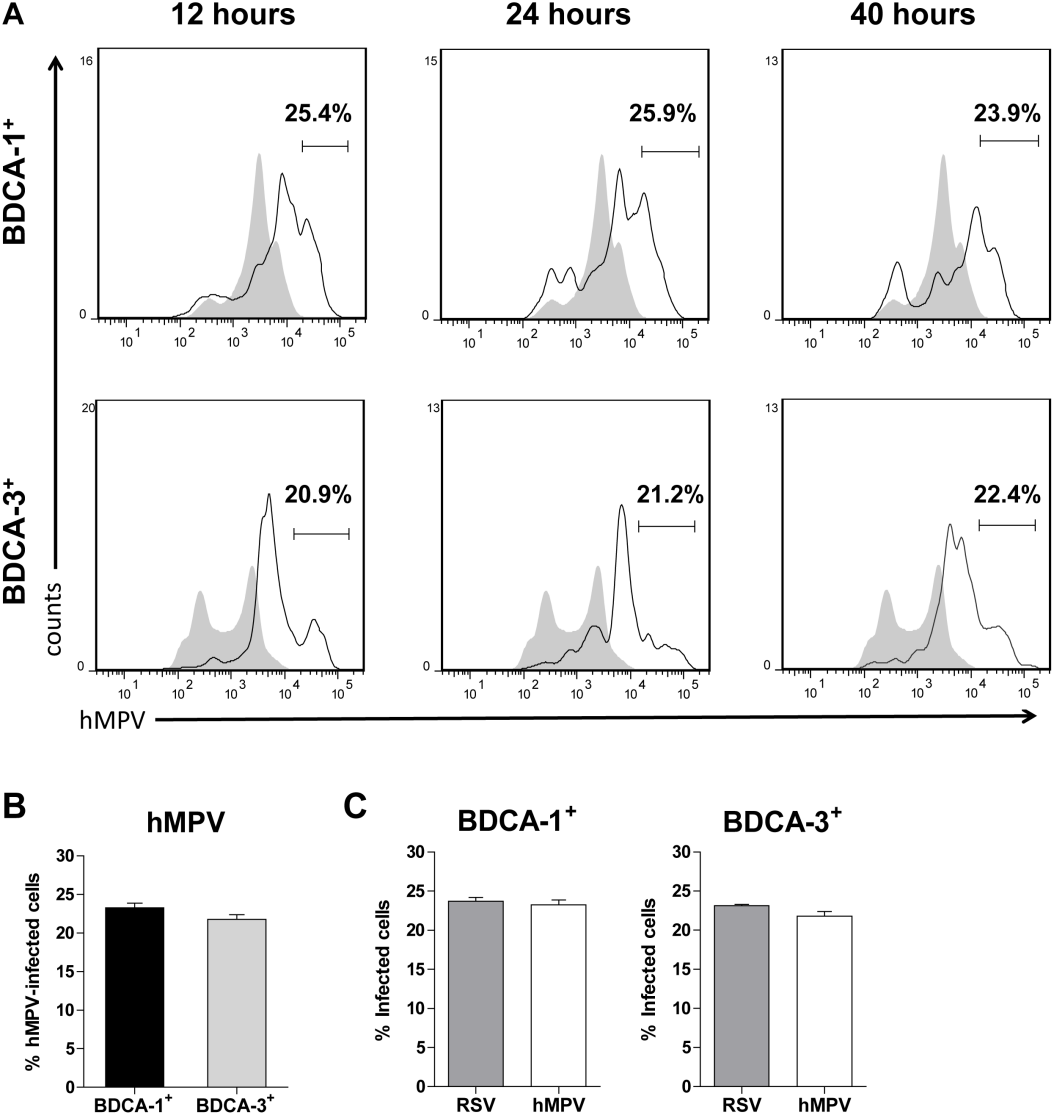
BDCA-1^+^ and BDCA-3^+^ mDCs are susceptible to infection with hMPV. (**A**) Kinetics of hMPV infection. BDCA-1^+^ and BDCA-3^+^ mDCs were exposed to hMPV at a MOI = 5 for 12, 24, or 40 hours. The percentage of live cells expressing anti-hMPV antibody was quantified using flow cytometry. Filled grey curve = uninfected DCs, solid line = hMPV-infected DCs,. Data is from one donor and is representative of three donors. (**B**) Comparison of hMPV infection rates in BDCA-1**^+^** vs. BDCA-3**^+^** mDCs (mean + SEM of three donors). (**C**) Comparison of infection rates by RSV vs. hMPV in each subset (mean + SEM of three donors).

Work from our lab has shown that Mo-DCs are susceptible to infection with RSV and hMPV [32], and we have recently shown that BDCA-1^+^ and BDCA-3^+^ mDCs are permissive to infection with RSV, with maximal infection rates occurring at a MOI = 5 [31]. To determine the susceptibility of primary mDCs to infection with hMPV, the intracellular expression of viral proteins by live BDCA-1^+^ and BDCA-3^+^ mDCs infected with hMPV at a MOI = 5 for 12, 24, and 40 hours was examined using flow cytometry. An MOI of 5 was chosen to maintain consistency for comparisons between RSV and hMPV. Expression of viral protein by BDCA-1^+^ and BDCA-3^+^ mDCs infected with hMPV was detected at all time points, (Fig. 1A). As in Mo-DCs [32], the number of hMPV positive cells did not increase with longer incubation times, suggesting that while primary mDCs are susceptible to hMPV infection, viral replication is restricted. Cell viability did decrease between 24 and 40 hours; however, there was no significant difference in cell viability between infected and uninfected cells at any given time point (data not shown). When comparing the rates of hMPV infection between BDCA-1^+^ and BDCA-3^+^ mDCs, no significant difference was noted in the percentages of BDCA-1^+^ mDCs (22–25%) and BDCA-3^+^ mDCs (20–22%) expressing viral proteins (Fig. 1B). Additionally, there was no statistically significant difference between the percentages of RSV- and hMPV-infected cells (Fig. 1C). Although cell viability in RSV-infected mDCs was noted to be decreased as compared to hMPV-infected mDCs, these differences were not statistically significant (data not shown).

### hMPV and RSV Induce Distinct Patterns of Co-stimulatory Molecule Expression by BDCA-1^+^ and BDCA-3^+^ mDCs

**Figure 2:**
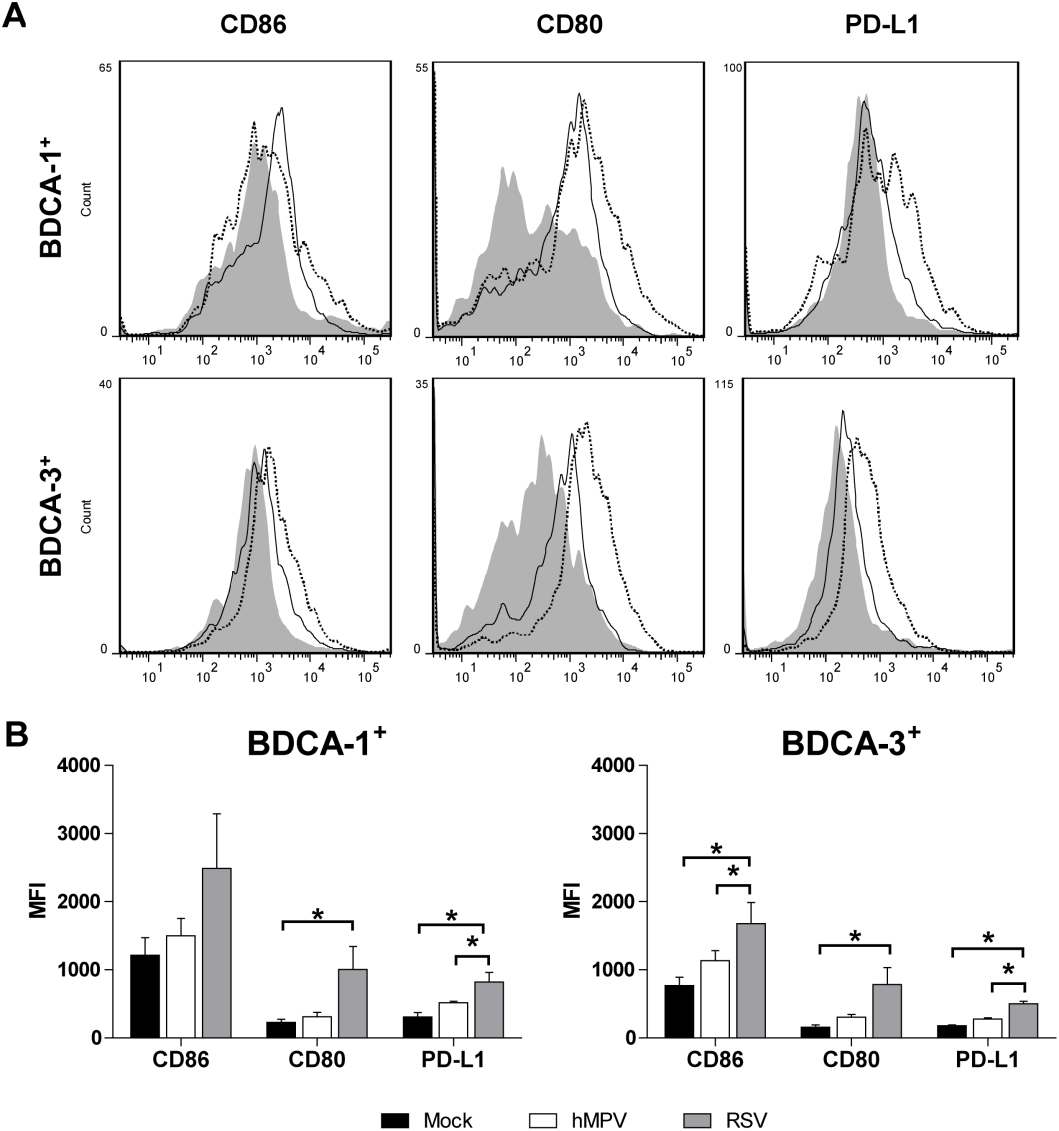
Virus induced expression of co-stimulatory molecules by BDCA-1^+^ and BDCA-3^+^ mDCs. (**A**) Co-stimulatory molecule expression on mDCs exposed to hMPV and RSV were analyzed by flow cytometry. Filled grey curve = uninfected DCs, solid line = hMPV-infected DCs, dotted line  =  RSV infected DCs. Data is from one donor and is representative of five donors. (**B**) Mean fluorescence intensity (MFI) of CD86, CD80, and PD-L1 expression by uninfected, RSV- and hMPV-infected mDCs from five donors (mean + SEM). Black bars = uninfected, white bars = hMPV, grey bars  =  RSV,. *p≤0.05 as calculated by 1-way repeated measures ANOVA with Tukey post-hoc analysis.

The co-stimulatory molecules expressed by DCs are critical in determining the fate of T cell activation and differentiation. CD86 and CD80 expressed on mDCs can deliver either stimulatory (CD28) or inhibitory (CTLA-4) signals for T cell activation [34]. Additionally, the programmed death-1 (PD-1) receptor and its ligands PD-L1 and PD-L2 deliver inhibitory signals that regulate the balance between tolerance vs. T cell activation and immune-mediated tissue damage. To determine the effect of hMPV infection on co-stimulatory molecule expression by primary mDCs and to assess whether RSV and hMPV differentially regulate co-stimulatory molecule expression, the expression of CD86, CD80, and PD-L1 on hMPV- and RSV-infected BDCA-1^+^ and BDCA-3^+^ mDCs was examined using flow cytometry (Fig. 2A). BDCA-1^+^ and BDCA-3^+^ mDCs infected with hMPV did not demonstrate significantly increased expression of CD86, CD80, and PD-L1 as compared to uninfected cells (Fig. 2B). Overall, RSV was a more potent inducer of CD80, CD86, and PD-L1 expression than hMPV, with significant differences in PD-L1 expression by RSV-infected BDCA-1^+^ mDCs, and CD86 and PD-L1 expression by RSV-infected BDCA-3^+^ mDCs (Fig. 2B).

### hMPV and RSV Induce Distinct Patterns of Cytokine and Chemokine Production by BDCA-1^+^ and BDCA-3^+^ mDCs

**Figure 3:**
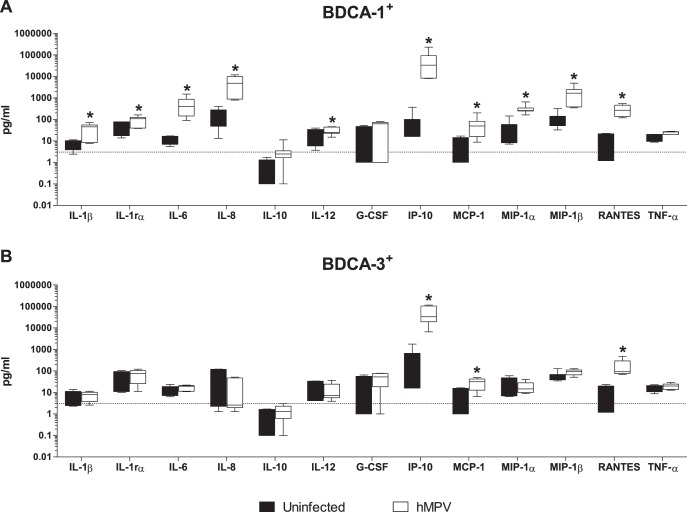
Cytokine production by hMPV-infected mDCs. (**A**) BDCA-1^+^ and (**B**) BDCA-3^+^ mDCs were isolated and incubated with hMPV or media for 40 hours. Cytokine and chemokine levels were measured in cell-free supernatant by multiplex assay. Data represent cytokine production in pg/ml. Box and whisker plots show the median (central bar), interquartiles (boxes), and range (whiskers) of the data from 6 donors. Linear data is reported on a logarithmic scale. Black bars = uninfected cells, white bars = hMPV-infected cells. Dotted line  =  threshold level of detection, 3 pg/ml. *p≤0.05 between infected and uninfected cells as calculated by paired t-test.

**Figure 4:**
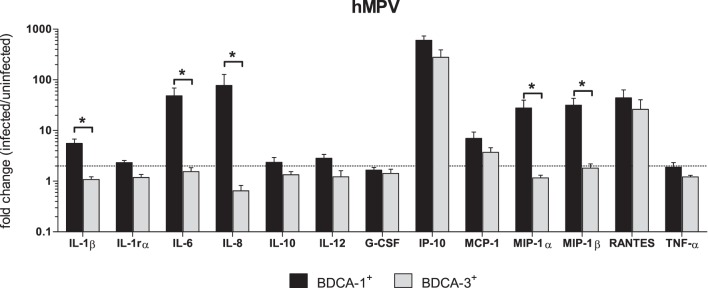
Comparison of selected cytokines produced by hMPV-infected BDCA-1^+^ vs. BDCA-3^+^ mDCs. DC subsets were isolated and incubated with hMPV or media for 40 hours. Cytokine and chemokine levels were measured in cell-free supernatant by multiplex assay. Data represents the mean + SEM of the fold change in cytokine production by infected cells as compared to uninfected cells of the same type from 6 donors. Linear data is reported on a logarithmic scale. The dotted line represents a 2 fold induction from baseline. Black bars = uninfected cells, white bars = hMPV-infected cells. *p≤0.05 between BDCA-1^+^ and BDCA-3^+^ mDCs as calculated by 2-way ANOVA with Bonferroni post-hoc test.

DC-derived cytokines and chemokines are at the center of the complex network of cells that regulate the outcome of innate and cellular immune responses. In both murine DCs and Mo-DCs, cytokine production is differentially induced by RSV and hMPV, with RSV being a more potent inducer of IL-10, TNF-α, IL-1β and IL-12 and hMPV being a more potent producer of IFN-α [32]. We have shown that RSV-infection induces differential cytokine production by BDCA-1^+^ and BDCA-3^+^ mDCs [31]. To define the profile of cytokines produced by primary mDC subsets in response to hMPV infection, cytokine production by hMPV-infected BDCA-1^+^ and BDCA-3^+^ mDCs was examined using a Luminex-based assay. Compared to uninfected cells of the same type, hMPV-infected BDCA-1^+^ mDCs demonstrated significant increases in the production of IL-1β, IL-1rα, IL-6, IL-8, IL-12, IP-10, MCP-1, MIP-1α, MIP-1β, and RANTES (Fig. 3A). In hMPV-infected BDCA-3^+^ mDCs, only IP-10, MCP-1 and RANTES levels were found to be significantly increased as compared to uninfected cells of the same type (Fig. 3B). Neither BDCA-1^+^ or BDCA-3^+^ mDCs infected with hMPV were found to produce significant amounts of IL-10, G-CSF, or TNF-α. In order to determine whether hMPV-infection induced distinct patterns of cytokine expression from BDCA-1^+^ and BDCA-3^+^ mDCs, the fold change in cytokine production (infected/uninfected) by each cell type was compared (Fig. 4). A two-factor analysis of variance showed a significant effect of cell type on cytokine production in response to hMPV, [F(1,104) = 125.8, p<.00001], as well as a significant effect of the cytokine being produced [F(12,104) = 37.44, p<.00001]. Overall, hMPV-infection induced greater amounts of cytokine production from BDCA-1^+^ than from BDCA-3^+^ mDCs. However, there was a significant interaction between cell type and cytokine, [F(12,104) = 7.77, p<.00001] suggesting that the effect of cell type was not the same on all cytokines, and compared to BDCA-3^+^ mDCs, hMPV-infected BDCA-1^+^ mDC were found to produce significantly greater amounts of IL-1β, IL-6, IL-8, MIP-1α, and MIP-1β. IL-1rα, IL-10, IL-12, G-CSF, IP-10, MCP-1, RANTES, and TNF-α production were not significantly different between the two cell types.

**Figure 5:**
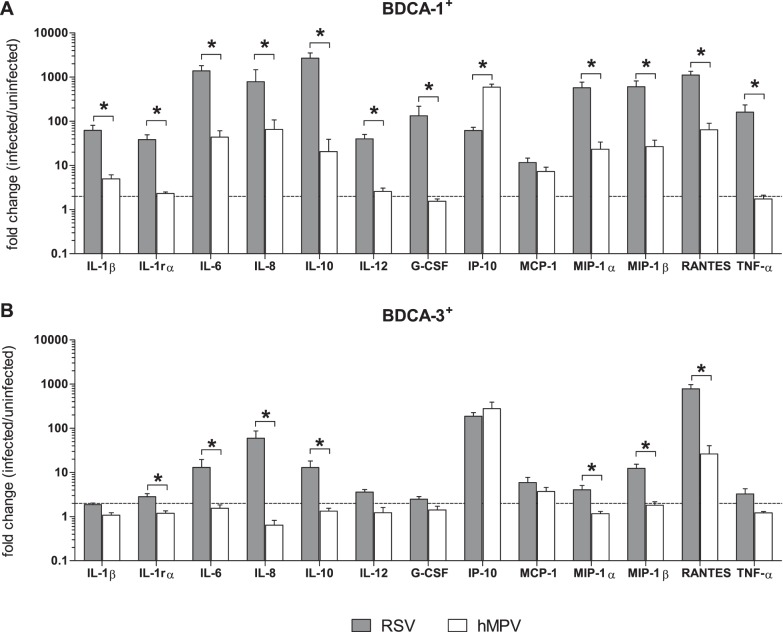
Comparison of cytokines produced by mDC subsets in response to RSV vs. hMPV. (**A**) BDCA-1^+^ and (**B**) BDCA-3^+^ mDCs were isolated and incubated with RSV, hMPV or media alone for 40 hours. Cytokine and chemokine levels were measured in cell-free supernatant by multiplex assay. Data represents the mean + SEM of the fold change (infected/uninfected) in cytokine production by RSV vs. hMPV-infected mDCs from 6 donors. Linear data is reported on a logarithmic scale. The dotted line represents a 2 fold induction. Grey bars = RSV-infected cells, white bars = hMPV-infected cells. *p≤0.05 between RSV and hMPV-infected mDCs as calculated by paired t-test.

Various reports in Mo-DCs and murine lung DCs have demonstrated impaired cytokine production by hMPV-infected DCs as compared to those infected with RSV [32], [35], [36]. To determine whether RSV and hMPV induce virus-specific patterns of cytokine expression, the fold change in cytokine production (infected/uninfected) by donor-matched RSV- and hMPV-infected cells was compared for each mDC subtype (Fig. 5). In general, RSV was found to induce more robust cytokine production from both BDCA-1^+^ and BDCA-3^+^ mDCs when compared to hMPV. With the exception of IP-10, produced in greater amounts by hMPV-infected BDCA-1^+^ mDCs, and MCP-1, which was not significantly different between RSV and hMPV infected BDCA-1^+^ mDCs, RSV-infected BDCA-1^+^ mDCs produced greater amounts of all cytokines tested (Fig. 5A). RSV-infected BDCA-3^+^ mDCs produced greater amounts of IL-1rα, IL-6, IL-8, IL-10, MIP-1α, MIP-1β, and RANTES. IL-1β, IL-12, G-CSF, IP-10, MCP-1, and TNF-α production by RSV and hMPV-infected BDCA-3^+^ mDCs was not significantly different (Fig. 5B).

**Figure 6:**
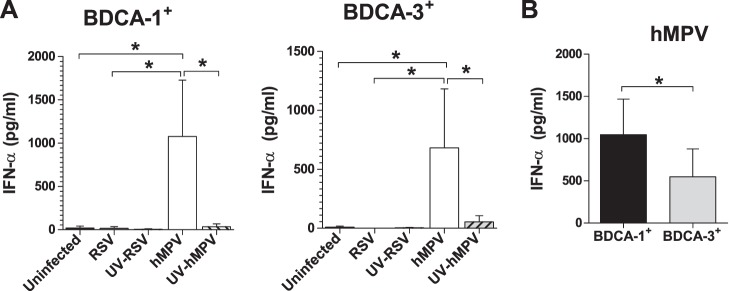
IFN-α production by RSV and hMPV-infected BDCA-1^+^ and BDCA-3^+^ mDCs. IFN-α production was assessed by ELISA in the supernatant of (**A**) BDCA-1**^+^** and (**B**) BDCA-3**^+^** mDCs incubated with RSV, hMPV, UV-RSV, UV-hMPV, or media for 40 hours (mean + SEM from 3 donors). *p≤0.05 between treated mDCs as calculated by repeated measures one-way ANOVA with Tukey post-hoc analysis. (**C**) Comparison of IFN-α production by hMPV-infected BDCA-1**^+^** vs. BDCA-3**^+^** mDCs (mean + SEM from 3 donors). * = *p≤0.05* between BDCA-1**^+^** and BDCA-3**^+^** mDCs as calculated by paired t-test.

Type 1 interferons (IFNs) are a group of cytokines that activate an array of cellular genes critical in restricting viral replication and modulating adaptive immunity, and the production of IFNs is an important feature of the host response to viral infections [37], [38]. In Mo-DCs, hMPV, but not RSV, is a potent inducer of IFN-α production [32]. To assess the ability of primary mDCs to produce IFN-α in response to hMPV infection, the production of IFN-α by RSV- and hMPV-infected BDCA-1^+^ and BDCA-3^+^ mDCs was measured using ELISA. In both BDCA-1^+^ and BDCA-3^+^ mDCs, IFN-α was produced by hMPV-infected cells, but not RSV-infected cells (Fig. 6A). As expected, IFN-α was not produced by mDCs exposed to UV-hMPV indicating that IFN-α production by mDCs is dependent on viral replication. When comparing IFN-α production between subsets, hMPV-infected BDCA-1^+^ mDCs produced greater amounts of IFN-α than did hMPV-infected BDCA-3^+^ mDCs (Fig. 6B).

### Differential Effect of hMPV and RSV Infection on the Ability of mDCs to Stimulate CD4^+^ T Cell Proliferation

**Figure 7:**
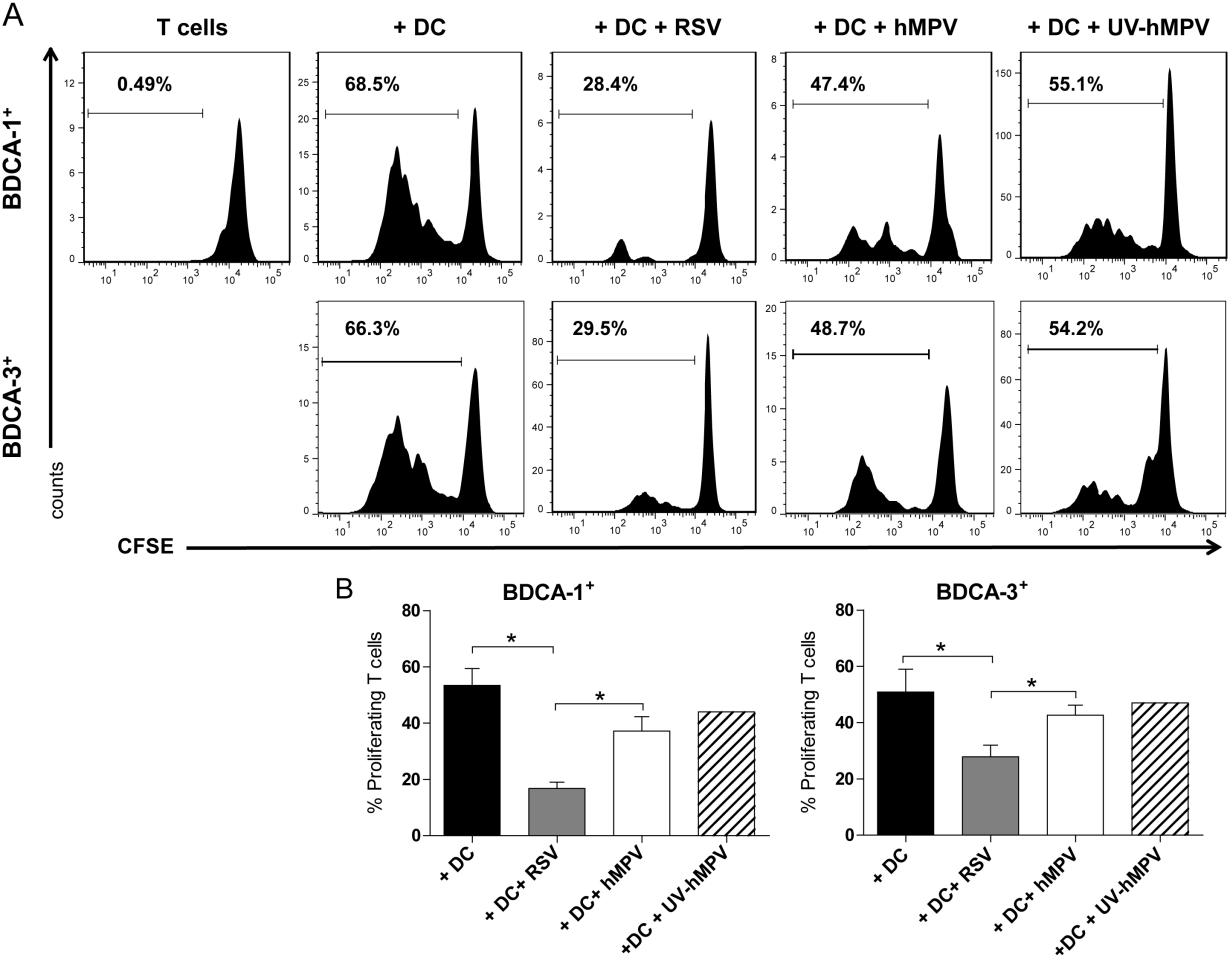
RSV, but not hMPV, impairs the ability of BDCA-1^+^ and BDCA-3^+^ mDCs to stimulate CD4^+^ T cell proliferation. BDCA-1^+^ and BDCA-3^+^ mDC were incubated with RSV, hMPV, UV-hMPV, or media for 24 hours and co-cultured with CFSE-labeled allogeneic CD4^+^ T cells. On day 7, T cell proliferation was measured by examining CFSE expression on live CD4^+^ T cells. (**A**) Data represent the percentage of proliferating cells from one donor and is representative of data from 3 donors. (**B**) Bar graphs represent the percent of proliferating CD4^+^ T cells (mean + SEM from 3 donors). * = *p≤0.05* between co-cultures with treated mDCs as calculated by one-way repeated-measures ANOVA with Tukey post-hoc analysis.

BDCA-1^+^ and BDCA-3^+^ mDCs are powerful and equivalent stimulators of allogeneic CD4^+^ T cells [24]. We have previously demonstrated that infection with RSV impairs the capacity of primary mDCs to stimulate T-cell proliferation [31]. To assess the effect of hMPV infection on the ability of BDCA-1^+^ and BDCA-3^+^ mDCs to stimulate T cell proliferation, allogeneic CD4^+^ T cells labeled with CFSE were co-cultured with BDCA-1^+^ and BDCA-3^+^ mDCs exposed to hMPV, RSV, UV-hMPV and UV-RSV for 7 days. BDCA-1^+^ and BDCA-3^+^ mDCs infected with hMPV demonstrated a reduced capacity to stimulate T cell proliferation as compared to uninfected mDCs or mDCs exposed to UV-hMPV (Fig. 7A). However, in contrast to RSV-infected mDCs, there was no statistically significant difference in the percent of proliferating T cells between co-cultures with hMPV-infected and uninfected mDCs (Fig. 7B).

### Differential Effect of hMPV and RSV Infection on Skewing of Th Polarization by Infected BDCA-1^+^ and BDCA-3^+^ mDCs

**Figure 8:**
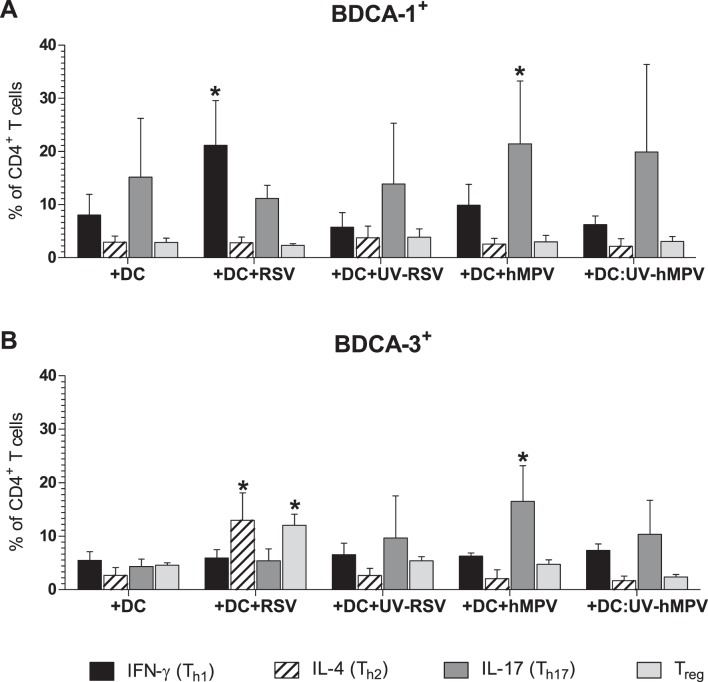
Skewing of CD4^+^ T cell polarization by virus-infected mDCs. Allogeneic CD4^+^ T cells were co-cultured with (**A**) BDCA-1^+^ and (**B**) BDCA-3^+^ mDCs incubated with RSV, hMPV, UV-RSV, UV-hMPV, or media. The percentage of live CD4^+^ T cells positive for expression of IFN-γ (Th1), IL-4 (Th2), IL-17A (Th17), and CD25^+^, Foxp3 (Tregs) was quantified using flow cytometry (mean + SEM from three donors). *p≤0.05 between the percentage of positive T cells from co-cultures with treated mDCs vs. co-cultures with uninfected DCs as calculated by one-way repeated-measures ANOVA with Tukey post-hoc analysis.

**Figure 9:**
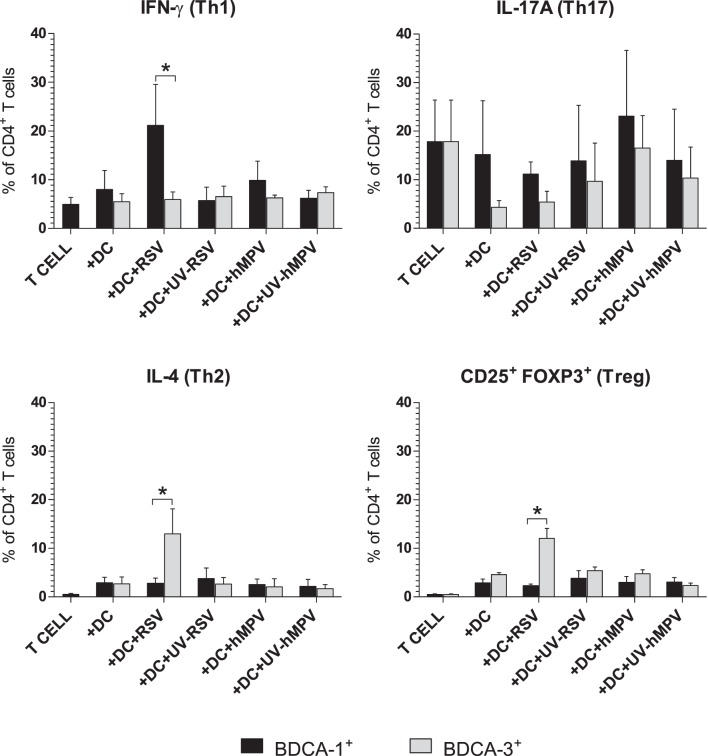
Comparison of Skewing of CD4^+^ T cell polarization by BDCA-1^+^ vs. BDCA-3^+^ mDCs. The percentage of each Th subset in T cell co-cultures with infected and uninfected BDCA-1^+^ vs. BDCA-3^+^ mDCs. *p≤0.05 between co-cultures with BDCA-1**^+^** and BDCA-3**^+^** mDCs as calculated by paired t-test.

The balance of the T helper subsets induced during infection plays a crucial role in determining the short- and long-term sequela of disease. To assess the effect of RSV and hMPV infection on the capacity of BDCA-1^+^ and BDCA-3^+^ mDCs to skew T cell polarization, allogeneic CD4^+^ T cells were co-cultured with mDCs exposed to RSV, hMPV, UV-RSV, or UV-hMPV. On day 8 post-infection, cells were collected and stained for extracellular expression of CD4 and CD25, and intracellular expression of IFN-γ, IL-4, IL-17A, and Foxp3. Cells were also stained with a fixable viability dye to discriminate between live and dead cells. The expression of IFN-γ, IL-4, IL-17A, and CD25 and Foxp3 was examined on live CD4^+^ T cells to identify Th1 (IFN-γ^+^), Th2 (IL-4^+^), Th17 (IL-17A^+^), and Treg (CD25^+^, Foxp3^+^) populations respectively (Fig. S1A–B). As expected, there were decreased numbers of live T cells in the co-cultures with RSV-infected mDCs as compared to co-cultures with hMPV-infected mDCs. This is likely due to the suppressive effect of RSV-infection on T cell proliferation, as there was no statistically significant difference in the percent of dead CD4^+^ T cells between co-cultures (data not shown). Compared to co-cultures with uninfected BDCA-1^+^ mDCs, there was an increased percentage of IFN-γ^+^ T cells in co-cultures with RSV-infected BDCA-1^+^ mDCs and an increased percentage of IL-17A^+^ T cells in co-cultures with hMPV-infected BDCA-1^+^ mDCs (Fig. 8A). There was no difference in the percent of IL-4^+^ or CD25^+^, Foxp3^+^ T cells between co-cultures with infected and uninfected BDCA-1^+^ mDCs. Compared to co-cultures with uninfected BDCA-3^+^ mDCs, co-cultures with hMPV-infected BDCA-3^+^ mDCs had an increased percentage of IL-17A^+^ T cells, and co-cultures with RSV-infected BDCA-3^+^ mDCs were found to have increased percentages of IL-4^+^ and CD25^+^, Foxp3^+^ T cells (Fig. 8B). For both BDCA-1^+^ and BDCA-3^+^ mDCs, the percentages of Th subsets in co-cultures with mDCs exposed to UV-inactivated RSV and hMPV were not significantly different than those found in co-cultures with uninfected mDCs. When comparing the Th types induced by BDCA-1^+^ mDCs to those induced by BDCA-3^+^ mDCs, RSV-infected BDCA-1^+^ mDCs were found to induce a greater percentage of IFN-γ^+^ T cells, whereas RSV-infected BDCA-3^+^ mDCs induced a greater percentage of IL-4^+^ and CD25^+^, Foxp3^+^ T cells (Fig. 9). There was no difference in the percentage of IFN-γ^+^, IL-4^+^, IL-17A^+^, or CD25^+^, Foxp3^+^ T cells induced by hMPV-infected BDCA-1^+^ and BDCA-3^+^ mDCs.

## Discussion

Both RSV and hMPV infection have been associated with aberrant adaptive T cell responses [11], [12], [14], [17], [19], and DCs play a central role in the induction and regulation of virus-specific adaptive immune responses in the respiratory tract. Although Mo-DCs infected with RSV or hMPV have not been found to skew T cells away from the Th1 compartment [39], phenotypic and functional differences between BDCA-1^+^ and BDCA-3^+^ mDCs suggest that each subset plays a distinct role in coordination of immune responses during infection, and studies in both mice [40]–[42] and humans [43]–[45] demonstrate a specific role for BDCA-3^+^ mDCs in the development of Th2 and Treg responses. In this study, we demonstrate a virus-specific and subset-specific effect of RSV and hMPV infection on the regulation of CD4^+^ T cell responses by primary mDCs. We show that similar to Mo-DCs [32], [35], [46], [47], BDCA-1^+^ and BDCA-3^+^ mDCs are susceptible to infection with RSV and hMPV and that infection with RSV, but not hMPV, impaired the capacity of primary mDCs to stimulate T cell proliferation. We also provide novel evidence demonstrating that in co-cultures with allogeneic CD4^+^ T cells, RSV-infected BDCA-1^+^ preferentially induced expansion of Th1 cells, whereas RSV-infected BDCA-3^+^ induced expansion of the Th2 and Treg compartments. Moreover, polarization of T cells away from Th1 responses by RSV-infected BDCA-3^+^ mDCs was also virus-specific, as both BDCA-1^+^ and BDCA-3^+^ mDCs infected with hMPV induced Th17 cell expansion.

Both the type and the strength of the co-stimulatory molecule signals produced by activated DCs determine the outcome of immune responses [48], and the differential expression of PD-L1 by RSV-infected BDCA-1^+^ and BDCA-3^+^ mDCs suggests a possible mechanism by which RSV infection could impair the ability of mDCs to stimulate T cell proliferation. Similar to findings in Mo-DCs [32], [35], even though hMPV was not found to be a strong inducer of co-stimulatory molecule expression, hMPV-infected mDCs did not inhibit T cell proliferation. The reason for this is unclear. Comparable infection rates by RSV and hMPV suggest that differences in infectivity did not lead to decreased mDC activation. However, it is not known what threshold level of co-stimulatory molecule expression is needed for T cell activation. Moreover, paracrine signaling by infected DCs has been shown to activate uninfected DCs [49], and the more robust production of cytokine signals provided by RSV-infected mDCs may have resulted in a greater number of activated cells.

RSV and hMPV infection have been shown to induce differential patterns of cytokine production in both *in* *vitro* and *in* *vivo* models of infection [19], [36], [50]–[52]. In line with these observations, RSV and hMPV induced virus-specific and subset-specific patterns of cytokine production from primary mDCs. Notably, IL-10 was produced only by RSV-infected mDCs of both subtypes. DC-derived IL-10 is shown to act in an autocrine fashion to inhibit pro-inflammatory cytokine production and impair the capacity of DCs to foster Th1 responses [53]. Moreover, only hMPV was a potent inducer of IFN-α production by BDCA-1^+^ and BDCA-3^+^ mDCs. Type 1 IFNs, such as IFN-α, potentiates antiviral activity through direct or indirect regulation of the activity of a number of proinflammatory cytokines and chemokines [37], [38], [54], promotes T cell proliferation, and inactivates the suppressive function of human Tregs, thereby releasing CD4^+^ T cells from Treg mediated suppression [55]. Additionally, we have previously shown that RSV induces subset-specific patterns of cytokine production [31], with differential production of IL-12 and TNF-α by RSV-infected BDCA-1^+^ mDCs. In contrast, although hMPV-infected BDCA-1^+^ mDCs produced greater amounts of cytokines than did BDCA-3^+^ mDCs, IL-12 and TNF-α production was not significantly different between the two subtypes. Pro-inflammatory cytokines such as IL-12, IFN-γ, and TNF-α are recognized as Th1 polarizing signals [56], [57]. Furthermore, IL-12 has been shown to enhance activation and proliferation of conventional T cells [58] and reduce the frequency and proliferation of Tregs [59]. In the periphery, inducible Tregs are derived from conventional T cells in the context of signals such as IL-2, IL-10, TGF-β, CD80/CD86, and PD-L1 [60]–[62]. Differential expression of CD86 by RSV-infected BDCA-3^+^ mDCs with the concomitant induction of IL-12 and other pro-inflammatory cytokines by RSV-infected BDCA-1^+^ mDCs, may explain the subset-specific effect of BDCA-3^+^ mDCs on Treg expansion during RSV infection. The cytokine signature associated with Th2 skewing is not as clearly defined; however, along with IL-4 and IL-10, low levels of IL-12 have been identified as Th2 polarizing signals [56], [57], [63]. Although RSV-infected BDCA-3^+^ mDCs were not found to produce IL-4, Th2 polarizing signals may have come from the activation and expansion of Th2 memory T cells in T cell co-cultures [64]–[66]. These cells could represent either direct activation of rare RSV-specific memory T cells or bystander activation of cross-reacting memory T cells. As Type 1 IFNs drive Th1 development while suppressing Th2 development [67], [68], differential expression of IFN-α and the absence of CD86, PD-L1 or IL-10 expression by hMPV-infected BDCA-3^+^ may explain the virus-specific effect of RSV and hMPV on T cell polarization by BDCA-3^+^ mDCs. Th17 cells develop under the influence of IL-1β, IL-6 and TGF-β, but have been shown to readily convert to a Th1 phenotype under inflammatory conditions [69] and recent evidence suggests that Th1 and Th17 cells may collaborate in the immune response against certain pathogens where Th17 cells precede and potentiate a Th1 response [70]. Th17 expansion in T cells co-cultured with hMPV-infected BDCA-1^+^ and BDCA-3^+^ mDCs may have resulted from the lack of adequate pro-inflammatory cytokine signals from hMPV-infected mDCs *in* *vitro*. Thus, although RSV-infected mDCs generally produced greater amounts of cytokines than hMPV-infected mDCs, lower levels of pro-inflammatory cytokines have not been found to correlate with decreased disease severity in patients with acute hMPV infection [50], suggesting that not only do RSV and hMPV utilize distinct pathways to modulate anti-viral immune responses, but that mDC subset-specific responses may be an important factor in determining the outcome of infection and the severity of clinical manifestations in RSV infection.

The mechanisms driving the differential responses induced by RSV and hMPV are unclear. Differences in their genomic structure, as well as differences in the way each virus interacts with DCs could contribute to the virus-specific responses. The NS1 and NS2 proteins of RSV inhibit production of IFN-α and drive production of pro-inflammatory cytokines through activation of the transcription factor NF-κB [11], [71], [72], and work from our lab has shown that the small hydrophobic glycoprotein (SH) of hMPV inhibits NF-κB dependent gene transcription [73]. Alternatively, activation of the host defense response is dependent on the recognition of conserved structural moieties by pattern recognition receptors (PRR), such as Toll-like receptors (TLRs), present on immune cells. TLRs convert pathogen recognition to active cellular responses through a complex network of intracellular molecules leading to the activation of transcription factors collaborating with each other to produce a large number of cytokines and co-stimulatory molecules, and the combination of signals produced in response to infection is dependent on the TLRs activated. Both RSV and hMPV have been shown to activate TLR4 in primary immune cells [74]–[77]; however, RSV has also been shown to mediate host immune responses through TLR3 dependent-signaling pathways [75], [78]–[80]. Moreover, BDCA-1^+^ and BDCA-3^+^ mDCs have been shown to express discreet TLR profiles [24], [81], [82] and differences in the way RSV interacts with each mDC subset may also underlie the subset-specific effect of RSV on mDC function. Further study to elucidate the mechanisms by which these viruses interact with BDCA-1^+^ and BDCA-3^+^ mDCs is needed.

To our knowledge, this is the first study to describe the functional response of primary mDCs to infection with hMPV, and also to compare their response to the two viruses. We recognize that the use of mixed lymphocyte reaction assays may not address antigen-specific responses that occur during natural infection. However, as it is not known whether RSV infection impairs the antigen presenting capacity of mDCs, the use of allogeneic T cells allowed us to focus on the functional activity of RSV-infected mDCs, independent of the quality or quantity of antigen presentation. Additionally, the use of total CD4^+^ T cells in co-culture experiments does not allow us to differentiate between Th subsets that arose from differentiation of naïve T cells versus the conversion or expansion of differentiated T cells as part of a recall response. However, It is not known what role recall responses play during acute infection as although more that 95% of adults have been exposed to RSV during childhood, there is a low frequency of RSV-specific memory T cells in the peripheral blood and lungs of healthy adults [83]. Moreover, examination of the frequency of memory and naïve T cell populations in CD4^+^ T cell isolates revealed that the majority of the CD4^+^ T cells in the peripheral blood samples used were naïve CD45ra^+^ T cells (34% CD45ra^+^ vs. 17% CD45ro^+^, data not shown). We recognize that using a reductionist *in* *vitro* model does not represent the interactions between the complex network of mDC subsets in the lungs and blood during natural infection and a suitable human model to further study these questions is needed. Furthermore, although RSV primarily affects young children, mDC subsets were isolated from the peripheral blood of healthy adults due to the feasibility of obtaining sufficient volumes of blood from pediatric populations. It is not well known whether age dependent differences in mDC subset function exist [84].

Despite these limitations, the results of this study provides novel information on the virus-specific and subset-specific role mDC subsets play in the immune response to *Paramyxovirus* infection. These findings suggest that despite the similarities in their clinical and immunopathologic features, RSV and hMPV utilize different pathways to modulate the host immune responses during infection, and that the quality and quantity of individual mDC subsets during RSV infection plays a crucial role in determining the short- and long-term sequela of disease. It is not known what type of mDCs are present in the lungs during natural infection, or how host genetic and environmental factors affect the downstream function of BDCA-1^+^ and BDCA-3^+^ mDCs interacting with virus. Defining the mechanisms by which RSV and hMPV are able to modulate mDC function will provide important new insights into the immunopathogenesis of paramyxovirus infections and the role mDC subsets play in the host immune response against pathogens as a whole.

## Supporting Information

Figure S1 **Identification of Th subsets in T cell co-cultures with virus-infected mDCs.** Allogeneic CD4^+^ T cells were co-cultured with **(A)** BDCA-1^+^ and **(B)** BDCA-3^+^ mDCs incubated with RSV, hMPV, UV-RSV, UV-hMPV, or media. The percentage of live CD4^+^ T cells positive for expression of IFN-γ (Th1), IL-4 (Th2), IL-17A (Th17), and CD25^+^, Foxp3 (Tregs) was quantified using flow cytometry. Data is from one donor and is representative of three donors.
(PNG)
