# Supplementary figures and images for: Paramyxovirus Infection Regulates T Cell Responses by BDCA-1+ and BDCA-3+ Myeloid Dendritic Cells
Source: PLoS One. 2014 Jun 11;9(6):e99227. doi: 10.1371/journal.pone.0099227 (PMC4053357; doi:10.1371/journal.pone.0099227)

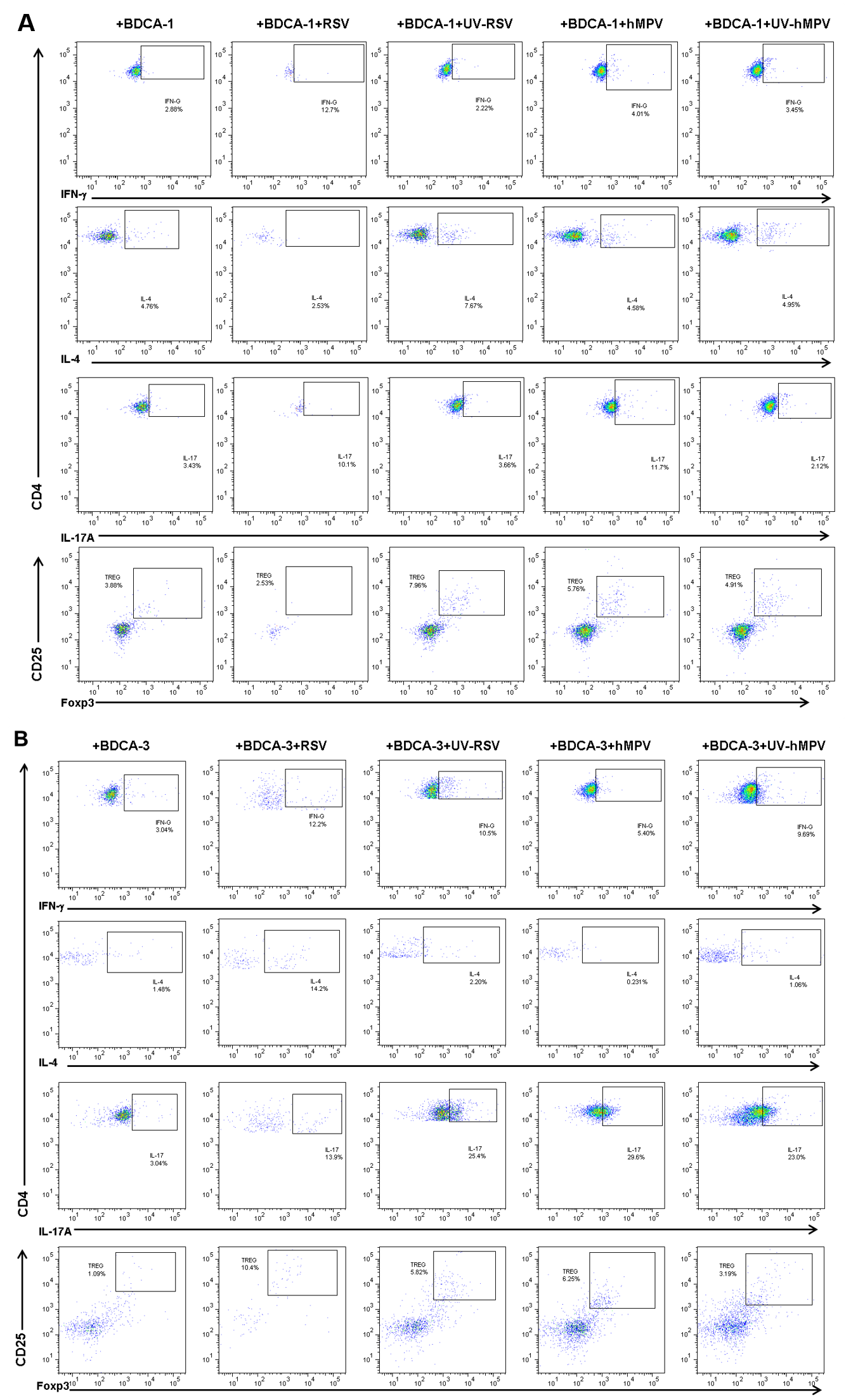

Supplement: Figure S1 — Identification of Th subsets in T cell co-cultures with virus-infected mDCs. Allogeneic CD4+ T cells were co-cultured with (A) BDCA-1+ and (B) BDCA-3+ mDCs incubated with RSV, hMPV, UV-RSV, UV-hMPV, or media. The percentage of live CD4+ T cells positive for expression of IFN-γ (Th1), IL-4 (Th2), IL-17A (Th17), and CD25+, Foxp3 (Tregs) was quantified using flow cytometry. Data is from one donor and is representative of three donors. (PNG) [file pone.0099227.s001.png]
